# Overnutrition in the Elderly Population: Socio-Demographic and Behavioral Risk Factors in Hungary

**DOI:** 10.3390/nu17121954

**Published:** 2025-06-08

**Authors:** Battamir Ulambayar, Amr Sayed Ghanem, Attila Csaba Nagy

**Affiliations:** 1 Department of Health Informatics, Faculty of Health Sciences, University of Debrecen, 4032 Debrecen, Hungary; ulambayar.battamir@etk.unideb.hu (B.U.); aghanem@etk.unideb.hu (A.S.G.); 2 Coordinating Centre for Epidemiology, University of Debrecen Clinical Centre, 4032 Debrecen, Hungary

**Keywords:** overnutrition, elderly population, eating habits, lifestyle factors, socio-economic factors

## Abstract

Background/Objectives: Overnutrition, leading to overweight and obesity, is a growing concern among the elderly, contributing to non-communicable diseases. This study examines socio-demographic, dietary, and lifestyle factors associated with overnutrition in Hungarian adults aged 65 and older. Methods: A cross-sectional analysis was conducted using 2019 European Health Interview Survey data, including 1628 elderly participants. Body mass index (BMI ≥ 25 kg/m^2^) defined overnutrition. Socio-demographic (gender, income, urbanization, partner status), dietary (fruit, vegetable, water, sweetener, salt intake), and lifestyle (alcohol, smoking, physical activity) factors were analyzed. Chi-square tests and multivariate logistic regression identified associations, with odds ratios (ORs) and 95% confidence intervals (CIs) calculated. Results: Overnutrition prevalence was 72.7%, higher in males (77.8%) than females (69.1%). Urbanization, income, and partner status showed associations. Significant predictors included lower water intake (OR = 0.47, 95% CI: 0.33–0.65 for 1–1.5 L), artificial sweetener use (OR = 1.54, 95% CI: 1.13–2.11), moderate/high salt intake (OR = 1.45, 95% CI: 1.06–1.99), former/never smoking (OR = 2.56, 95% CI: 1.73–3.77), and heavy alcohol use (OR = 4.00, 95% CI: 1.33–12.50). Conclusions: Artificial sweetener use, high salt intake, smoking history, and heavy alcohol consumption are key modifiable predictors of overnutrition, informing targeted interventions for elderly Hungarians.

## 1. Introduction

Overnutrition, characterized by excessive nutrient intake leading to overweight (BMI ≥ 25) or obesity (BMI ≥ 30), is a growing public health concern, contributing to non-communicable diseases (NCDs) such as type 2 diabetes, cardiovascular disease, and metabolic syndrome [1]. Globally, over 1.9 billion adults are overweight or obese, with elderly populations increasingly affected due to longer life expectancies and lifestyle changes [2]. In Europe, obesity prevalence exceeds 20%, with Central and Eastern European nations facing higher rates due to post-socialist dietary shifts [3]. These trends impose significant economic burdens, with obesity-related NCDs costing billions annually [4]. Beyond physical health, overnutrition impairs mental well-being [5], increasing anxiety and depression among those struggling with weight issues [6].

The aging global population presents unique health challenges, exacerbated by the rising prevalence of overnutrition among older adults [7]. Overnutrition is a significant problem among young people, primarily driven by lifestyle factors [8]. However, aging introduces physiological changes, such as reduced metabolic rate and sarcopenia, which predispose older adults to weight gain [9]. Psychological factors, including loneliness and bereavement, may drive emotional eating, particularly in socially isolated individuals [10]. Limited access to exercise facilities or safe outdoor spaces further restricts physical activity [11].

Historically, public health focused on undernutrition in the elderly, but overnutrition now demands equal attention [12]. Urbanization and globalization expose older adults to high-calorie food choices, increasing obesity risk [13]. This demographic also faces a double burden, with some experiencing concurrent obesity and micronutrient deficiencies or sarcopenic obesity, complicating healthcare delivery by requiring integrated nutritional screening [12,13].

Overnutrition is shaped by socio-cultural, economic, and behavioral factors beyond diet [14]. Economic resources influence nutritional choices, with higher incomes linked to greater dietary diversity and quality, potentially mitigating overnutrition risk [15]. Conversely, low-income individuals rely on cheap, high-calorie processed foods [16]. Urban environments, with abundant fast food, contribute to overnutrition [15,16]. Gender and education also play roles [17,18], with women more vulnerable due to hormonal changes and caregiving demands, and higher education correlating with better nutritional knowledge [19]. Sedentary lifestyles and irregular eating patterns, common in the elderly, exacerbate risks [20]. Structural factors, such as high healthy food prices or poor urban walkability, further limit healthier options [21].

In Hungary, where over 30% of adults are obese and the population is rapidly aging, obesity-related diseases pose a significant health burden and elevate mortality risk [22]. Traditional high-fat diets and post-socialist shifts toward processed foods exacerbate this issue [23]. Hungary’s healthcare system struggles with limited preventive programs, amplifying mortality risks [24]. Existing interventions, such as dietary guidelines, often fail to address elderly-specific needs [25], and research on socio-demographic predictors in Central Europe remains limited [23,24].

A comprehensive approach, incorporating personalized dietary modifications, promotion of physical activity, and lifestyle interventions, is essential for promoting healthy aging [26,27]. Accordingly, this study examines the relationships between socio-demographic, dietary, and lifestyle factors and overnutrition among older adults in Hungary, employing logistic regression to identify predictors for targeted interventions.

## 2. Materials and Methods

### 2.1. Study Design and Data

This research employed a cross-sectional approach, utilizing data gathered in Hungary during 2019 as part of the European Health Interview Survey (EHIS). The data were collected under Eurostat’s oversight with a standardized questionnaire, with face-to-face and the sample (*n =* 5603) was adequately *representative* of Hungary’s general population. Participation in the EHIS was voluntary. While reasons for non-participation are not documented, possible barriers include poor health, cognitive impairment, or institutionalization, which may have introduced selection bias. Data collection was conducted through face-to-face interviews by trained interviewers using a standardized questionnaire, allowing participants to seek clarification on any unclear questions [28]. From this sample, 1628 individuals aged 65 years or older were selected for inclusion and analysis in this study.

### 2.2. Variables

Anthropometric data were collected using standardized questions: “How tall are you without shoes?” and “What is your body weight without clothes and shoes?” If participants did not know or could not recall, trained interviewers performed measurements according to EHIS protocol using standardized equipment. Using height and weight data from older adults included in this study, body mass index (BMI) was calculated. A binary variable (outcome variable) was then established, categorizing individuals with a BMI of ≥25 kg/m^2^ as overweight or obese (indicative of overnutrition) and those with a BMI < 25 kg/m^2^ as normal weight, consistent with WHO guidelines [29].

The following variables, contained in the EHIS data, were selected as explanatory variables to influence overnutrition in the elderly. Socio-demographic variables included gender, education, household income level, degree of urbanization, and partner status and health-related variables comprised self-perceived health status, and presence of long-term illness. Dietary habits were assessed through weekly consumption frequencies of fruits, vegetables, fruit juice, sugary/soft drinks, sugar-free/diet drinks, sweets/desserts, red meat, white meat, processed meat, fish/seafood, and dairy products. Dietary frequency variables were categorized into daily, several times per week (4–6 times), and occasionally (less than 3 times per week) based on the distribution of responses in our dataset to distinguish between low, moderate, and high intake levels. This categorization aligns with established dietary guidelines for key food groups [30]. Daily water intake was recorded as ≥2 L, 1–1.5 L, or ≤1 L. Sweetener use for hot drinks (coffee, tea) was classified as natural (sugar, honey) or artificial, and salt consumption was dichotomized as never/low or moderate/high. Lifestyle factors included alcohol consumption (heavy, moderate, rare/never), smoking status (active, quit, never smoked), and physical activity. Physical activity was evaluated through general daily activity (mostly sitting/no movement, mostly standing, mostly walking/moderate, mostly heavy physical work), number of days per week with at least 10 min of walking, cycling, sports, or muscle-strengthening exercises, and sleep disturbances in the past two weeks.

### 2.3. Statistical Analysis

Data were analyzed using descriptive statistics to summarize participant characteristics, dietary habits, and lifestyle factors, stratified by BMI category. Frequencies and percentages were calculated for categorical variables, and chi-square tests were used to assess differences between BMI groups, with *p*-values indicating statistical significance (*p* < 0.05). Multivariate logistic regression was employed to investigate associations between overnutrition and socio-demographic, dietary, and lifestyle factors. The model included variables such as gender, income level, degree of urbanization, partner status, long-term illness, fruit and vegetable consumption, water intake, fruit juice intake, sweetener use, salt consumption, smoking status, alcohol use, and days of sports per week based on the significant associations detected by chi-square test. Odds ratios (ORs) with 95% confidence intervals (CIs) were calculated to estimate the strength of associations, with *p*-values < 0.05 considered significant. To evaluate the discriminative ability of the logistic regression model, we calculated the area under the receiver operating characteristic (ROC) curve (AUC). STATA IC version 18.0 was used to conduct the statistical analyses used in this analysis [31].

## 3. Results

The analysis of the study population, as presented in Table 1, provides an overview of the associations between socio-demographic and health-related factors and body weight status, categorized as normal BMI (<25) and overnutrition (BMI ≥ 25). The sample comprised 1628 participants, with a higher proportion of females (59.3%, *n =* 966) compared to males (40.7%, *n =* 662). A significant association was observed between gender and body weight status (*p* < 0.001), with males exhibiting a higher prevalence of overnutrition (77.8%) compared to females (69.1%). The prevalence of overnutrition was relatively consistent across education levels, ranging from 70.1% in the secondary education group to 74.3% in the primary education group. Household income level, divided into lower than average (47.0%, *n =* 766), average (23.5%, *n =* 382), and higher than average (29.5%, *n =* 480), demonstrated a statistically significant association with body weight status (*p* = 0.032). Participants with higher-than-average income had the lowest prevalence of overnutrition (68.7%), while those with lower-than-average income had the highest (75.4%). The degree of urbanization, categorized as urban (19.5%, *n =* 317), suburban (20.5%, *n =* 334), rural (32.6%, *n =* 530), and remote (27.4%, *n =* 447), was also significantly associated with body weight status (*p* = 0.038). Participants in urban areas had the lowest prevalence of overnutrition (68.7%), while those in remote areas had the highest (77.4%). Partner status, divided into living with a partner (53.5%, *n =* 858) and living without a partner (46.5%, *n =* 746), showed a significant association with body weight status (*p* = 0.001). Participants living without a partner had a lower prevalence of overnutrition (68.5%) compared to those living with a partner (76.3%). the presence of a long-term illness, reported by 77.1% (*n =* 1245) of participants, was significantly associated with body weight status (*p* = 0.015). Participants without a long-term illness had a lower prevalence of overnutrition (67.6%) compared to those with a long-term illness (74.0%).

Table 2 shows association between eating habits and body weight status among older adults. Fruit consumption per week was significantly associated with body weight status (*p* = 0.031). Participants consuming fruit less than three times a week had the lowest prevalence of overnutrition (65.9%), compared to those consuming fruit every day (74.0%) or 4–6 times a week (72.2%). Vegetable consumption (excluding potatoes) per week also showed a significant association with body weight status (*p* = 0.045). Participants consuming vegetables 4–6 times a week had the lowest prevalence of overnutrition (68.5%), compared to those consuming vegetables every day (75.1%) or less than three times a week (70.5%). Daily water consumption showed a highly significant association with body weight status (*p* < 0.001). Participants who consumed one liter or less of water per day had the lowest prevalence of overnutrition (64.3%), while those who consumed at least two liters had the highest (78.4%), suggesting that polydipsia associated with obesity and metabolic syndrome may contribute to increased water intake in these individuals. Fruit juice consumption per week was significantly associated with body weight status (*p* = 0.037). In contrast, participants consuming fruit juice 4–6 times a week had the lowest prevalence of overnutrition (62.7%), compared to those consuming it every day (66.7%) or less than three times a week (73.5%). The use of sweeteners for hot drinks (coffee, tea) showed a significant association with body weight status (*p* = 0.002). Participants with overnutrition use more artificial sweeteners (78.3%) compared to those without overnutrition (69.5%). Finally, salt consumption was significantly associated with body weight status (*p* = 0.038). Participants with moderate or high salt use had a higher prevalence of overnutrition (76.3%) compared to those with never or low salt use (71.1%).

The analysis of associations between lifestyle behaviors and body weight status among older adults presented in Table 3. Participants with heavy alcohol consumption exhibited the highest prevalence of overnutrition (85.5%), compared to those with moderate (73.2%) or rare/never consumption (71.8%). Smoking status demonstrated a highly significant association with body weight status (*p* < 0.001). Active smokers had the lowest prevalence of overnutrition (55.8%), while those who had quit smoking had the highest (78.1%), followed by those who never smoked (73.8%). The number of days per week performing sports for at least 10 min was also significantly associated with body weight status (*p* = 0.026). Participants engaging in sports 4–7 days a week had the lowest prevalence of overnutrition (65.1%), compared to those performing sports 1–3 days (73.2%) or not performing sports (74.0%).

The logistic regression analysis, as presented in Table 4, identifies significant predictors of overnutrition (BMI ≥ 25) among the elderly population in Hungary, focusing on socio-demographic, dietary, and lifestyle factors. Water intake was a significantly associated with overnutrition. Compared to individuals consuming more than 2 L of water daily, those consuming 1.5–2 L had a lower odds of overnutrition (OR = 0.68, 95% CI: 0.48–0.95, *p* = 0.025), and those consuming 1–1.5 L had an even lower odds (OR = 0.47, 95% CI: 0.33–0.65, *p* < 0.001). The use of artificial sweeteners for tea or coffee was also significantly associated with overnutrition. Compared to those using natural sweeteners, individuals using artificial sweeteners had higher odds of overnutrition (OR = 1.54, 95% CI: 1.13–2.11, *p* = 0.006). Salt use was a significant predictor. Individuals with moderate or high salt use had higher odds of overnutrition compared to those with never or low salt use (OR = 1.45, 95% CI: 1.06–1.99, *p* = 0.020). Smoking status was strongly associated with overnutrition. Compared to active smokers, individuals who had quit smoking had significantly higher odds of overnutrition (OR = 2.32, 95% CI: 1.49–3.61, *p* < 0.001), as did those who never smoked (OR = 2.56, 95% CI: 1.73–3.77, *p* < 0.001). Alcohol consumption was also a significant predictor. Compared to heavy drinkers, moderate drinkers had lower odds of overnutrition (OR = 0.26, 95% CI: 0.09–0.80, *p* = 0.019), as did rare drinkers (OR = 0.25, 95% CI: 0.08–0.75, *p* = 0.018). To explore potential confounding interactions, we conducted a logistic regression analysis including an interaction term between smoking status and alcohol consumption. However, no statistically significant interaction effect on body weight status was observed (*p* > 0.05).

## 4. Discussion

This study aimed to identify relationships between socio-demographic, dietary, and lifestyle factors and overnutrition among Hungarian adults aged 65 years and older. The findings revealed significant associations with gender, long-term illness, socio-economic factors (including income levels, degree of urbanization, and partner status), eating habits (including fruit and vegetable consumption, water and fruit juice intake, use of sweeteners and salt), and lifestyle factors (including alcohol consumption, smoking status, and sports activity). However, after adjusting for all significant variables in multivariate logistic regression, reduced water consumption, use of artificial sweeteners, moderate-to-high salt intake, smoking history (former or never smoked), and heavy alcohol use were significantly correlated with overnutrition among the elderly population in Hungary.

Research shows that the prevalence of overweight and obesity among older adults increases with age, and there are significant gender differences in the prevalence of overnutrition and its impact on health [17,32]. Older women are at increased risk of obesity-related health complications due to physiological changes after menopause [33]. Declining estrogen levels alter metabolic processes and lead to changes in the distribution of fat and lean mass [34]. In many cultures, women often play the primary caregiver role, which affects their diet and access to nutrition. Studies show that both sexes are vulnerable to malnutrition, but older women are more likely to be malnourished due to socio-economic constraints [35], which indirectly contributes to their obesity [36]. The higher prevalence of overnutrition among older Hungarian males compared to females, despite literature suggesting female vulnerability, may reflect Hungary-specific socio-cultural factors, such as dietary patterns and lifestyle risks.

The relationship between socio-economic factors and overnutrition in older adults highlights the complex dynamics that influence dietary patterns, health outcomes, and quality of life in older adults. Research suggests that socio-economic status is an important determinant of nutritional health and has a significant impact on the rate of overnutrition among the elderly. In some countries, wealthy older people tend to have better access to a variety of food options and consume higher-calorie foods, which contributes to the increase in obesity and related health problems [37]. Conversely, individuals from lower socio-economic backgrounds often encounter food insecurity and limited access to nutritious diets, which results in poorer nutritional quality overall [38]. However, being highly educated and having a sufficient income may have a positive impact on the nutritional status of the elderly. Higher levels of education are associated with health literacy, which influences food choices and nutritional awareness [39]. Housing and community factors related to socio-economic status play an important role in influencing health outcomes. Low socio-economic status is associated with reduced opportunities for social participation, which may exacerbate health inequalities among older adults. Social isolation, a common problem among socially disadvantaged groups, can hinder physical activity, which is important for weight control and maintaining health [40]. This is supported by our results that socio-economic variables such as income levels, degree of urbanization, and partner status have been found to be associated with overnutrition among the elderly.

Eating habits, such as meal frequency and the types of foods consumed, are directly related to calorie intake and, consequently, overeating. Studies have shown that older adults who frequently snack, especially those high in sugar and fat, have higher calorie intake than those who eat regularly [41]. Cultural considerations further shape the dietary habits of the elderly population. Research has shown that traditional dietary practices influence food choices and nutritional outcomes in older adults [42,43]. Hungarian traditional cuisine tends to be calorically dense, often featuring meat as the primary ingredient, complemented by hearty sides like potatoes, dumplings, and bread [44]. This characteristic of traditional cuisine may be one reason for the high prevalence of obesity among the elderly. Interestingly, most studies have shown that decreasing fruit and vegetable consumption increases the risk of obesity among older adults [45,46,47], yet our findings contradict this, with higher fruit juice consumption associated with a lower rate of overnutrition. This may be attributed to Hungary’s unique dietary patterns, differences in food preparation methods (vegetables cooked with added fats), and potential biases inherent in self-reported dietary data. Socio-economic factors, or compensatory nutritional habits that mitigate the expected effects of higher fruit and vegetable consumption, as these associations were not observed after adjusting for socio-economic and lifestyle variables in the logistic regression model. Additionally, reverse causation is possible—individuals with higher BMI may increase their fruit and vegetable intake in response to health advice, which could obscure causal relationships.

Our study results revealed that using artificial sweeteners increases overnutrition among older adults by 1.54 times. Most of studies show that these non-nutritive sweeteners contribute to health complications, including weight gain, same as our results. Although people with overnutrition may be more likely to use calorie-free sweeteners for weight loss, one prominent concern is that the use of artificial sweeteners may not effectively promote weight loss or better nutritional outcomes, which is often their intended purpose. Some evidence suggests that artificial sweeteners might paradoxically lead to increased caloric intake due to their effects on appetite and metabolism [48]. The intense sweetness from artificial sweeteners may lead to increased cravings for sweet foods, potentially resulting in overconsumption of both sweet and calorically dense foods [49]. Moreover, certain studies have indicated that those who consume artificially sweetened beverages might experience altered gut microbiota composition, which could predispose them to weight gain and insulin resistance [50]. The metabolic implications are particularly critical for the elderly, as many in this population may already struggle with maintaining a healthy weight due to various factors such as reduced physical activity, hormonal changes, and chronic health issues. Research has shown a correlation between high consumption of artificial sweeteners, such as aspartame and sucralose, and adverse metabolic outcomes, including an increased risk of T2D [51] and cardiovascular diseases [52]. Additionally, long-term studies have suggested that older adults who regularly consume artificially sweetened products may be unaware of the potential health risks associated with their intake. The increasing visibility of diet products marketed towards older adults often leads to a misconception that these items are inherently healthy, prompting frequent consumption [53]. While artificial sweeteners are often promoted as a solution for weight management, evidence suggests that their consumption may be linked to negative health outcomes that contribute to overnutrition in the elderly population. This underscores the need for further research and improved public awareness about the implications of artificial sweeteners on health, particularly among older adults who may rely on these products to manage dietary concerns.

Research indicates that excessive salt intake can contribute to several health concerns, including hypertension, obesity, and other metabolic disorders, particularly in older adults. High dietary salt is known to elevate blood pressure, which is a significant risk factor for cardiovascular diseases and is especially pertinent in the elderly due to age-related increases in vascular stiffness and other cardiovascular changes [54]. Moreover, there is growing evidence that excessive salt intake is associated with overweight and obesity. Studies have shown that high salt intake promotes fat storage and is positively correlated with BMI in older adults [55,56]. A study found a correlation between salt intake and obesity indicators, noting that excessive salt might contribute to fat accumulation and affect metabolic health [57]. High salt intake among the Hungarian population remains a major public health problem, and it is one of the main causes of overnutrition among the elderly, which is consistent with our study results. 

The associations between smoking status and overnutrition among elderly reveal significant implications for public health, particularly concerning dietary behaviors and health outcomes in this demographic. Research indicates that individuals categorized as “former smokers” and “never smoked” exhibit higher odds of overnutrition compared to active smokers. The observed odds ratios suggest a potential paradox where those who do not currently smoke may have dietary habits that contribute to obesity. This association is consistent with findings from previous literature. A systematic review explored smoking as a predictor of frailty among older adults, emphasizing how lifelong smoking behavior can influence overall health outcomes, and it supports broader findings that non-smokers or those who quit smoking may adopt different eating patterns, potentially leading to overnutrition following smoking cessation [58]. Interestingly, another study found that elderly smokers typically have lower BMI than non-smokers, suggesting that smoking may play a role in weight management through appetite suppression and increased energy expenditure [59]. This finding contrasts with the higher odds of overnutrition observed in former and never smokers, indicating that cessation of smoking or not smoking at all does not guarantee protective effects against obesity in the elderly. Moreover, Hsieh et al. underlined the necessity of considering confounding variables, including smoking status [60]. Further underscoring these interactions, Lee et al. showed that higher obesity levels were associated with significantly lower quality of life, emphasizing the relevance of smoking cessation in relation to dietary habits and obesity management among elderly populations [61]. Therefore, public health interventions encouraging smoking cessation must be paired with dietary education to help mitigate risks associated with obesity.

The findings indicate that moderate and rare drinkers exhibit significantly lower odds of overnutrition compared to heavy drinkers. This supports the hypothesis that patterns and amounts of alcohol consumption can influence nutritional statuses and overall health outcomes in older adults. Research suggests that light to moderate alcohol consumption may have protective effects against developing metabolic syndrome and its components, such as obesity, hypertension, and dyslipidemia [62], which is possibly linked to the lower odds of overnutrition found in our study. The effects of heavy alcohol consumption are compounded by the potential for developing nutritional deficiencies. Evidence has shown that alcohol can interfere with the metabolism of several key nutrients, leading to imbalances that intensify weight gain and negatively impact overall health [63].

Research indicates that moderate physical activity can enhance metabolism, facilitating better energy use and preventing the accumulation of excess body weight among the elderly [64]. Although unadjusted analysis suggested that higher frequency of sports activity was associated with a lower incidence of overnutrition, this association did not persist in the multivariable logistic regression. This indicates that other factors, such as age, comorbidity burden, or obesity, may mediate or confound the relationship between physical activity and health outcomes in this population. In older adults, barriers to regular physical activity are multifactorial and include physical limitations [65], psychosocial factors [66], and environmental obstacles [67].

Our results showed a significant association between higher water intake and overnutrition, which may reflect reverse causality rather than a direct effect. Older adults with overnutrition often consume more water due to increased caloric intake [68], higher sodium content in their diets [69], and greater metabolic demands associated with higher body mass [70]. Additionally, changes in body composition and fluid balance in obesity, as well as cognitive factors affecting thirst perception, may influence drinking behavior [71]. These considerations suggest that elevated water intake in the group with overnutrition may be a compensatory response to physiological and dietary factors rather than a driver of overnutrition.

This study has some limitations, including the use of self-reported questionnaires, which may lead to bias from inaccurate recall, social desirability, or misreporting. Participants might over- or underestimate their dietary intake and physical activity, causing measurement errors. The cross-sectional design also prevents determining causal relationships between overnutrition and related factors. However, a major strength is the application of advanced statistical methods, like logistic regression, to analyze reliable data collected through a Eurostat-validated questionnaire, which is representative of the Hungarian population.

## 5. Conclusions

The findings show significant links between lower water intake, artificial sweetener use, moderate-to-high salt intake, smoking status (non-smoker or former smoker), and heavy alcohol consumption with overnutrition in elderly Hungarians. However, water intake is unlikely to be a direct cause of overnutrition and may instead reflect metabolic changes and cognitive factors that drive increased consumption. In contrast, artificial sweetener use, high salt intake, heavy alcohol use, and smoking status emerged as strong, independent predictors of overnutrition, regardless of gender, socio-economic factors, diet, or physical activity, emphasizing their importance as modifiable lifestyle factors.

Our findings support routine screening and lifestyle counseling in nutritional care, particularly for older adults with limited physical activity, and highlight the need for public health strategies in Central and Eastern Europe that promote healthy lifestyles and weight management through community-based programs and improved access to age-appropriate resources. Future longitudinal and interventional studies are recommended to better understand causal relationships and to evaluate the effectiveness of specific public health strategies aimed at modifying these risk factors.

## Figures and Tables

**Table 1:** Demographic and health status data of participants.

Variable	Category	Total n (%)	Body Weight		*p* Value
			Normal BMI < 25	Overnutrition BMI ≥ 25	
Gender	Male	662 (40.7)	146 (22.2)	511 (77.8)	**<0.001**
	Female	966 (59.3)	296 (30.9)	661 (69.1)	
Education	Primary	830 (51.0)	212 (25.7)	612 (74.3)	0.312
	Secondary	496 (30.5)	143 (29.1)	349 (70.1)	
	High	302 (18.5)	87 (29.2)	211 (70.8)	
Household income level	Lower than average	766 (47.0)	187 (24.6)	575 (75.4)	**0.032**
	Average	382 (23.5)	107 (28.2)	272 (71.8)	
	Higher than average	480 (29.5)	148 (31.3)	325 (68.7)	
Degree of urbanization	Urban	317 (19.5)	98 (31.2)	215 (68.8)	**0.038**
	Suburban	334 (20.5)	91 (27.3)	242 (72.7)	
	Rural	530 (32.6)	153 (29.1)	372 (70.9)	
	Remote	447 (27.4)	100 (22.6)	343 (77.4)	
Partner status	Living with a partner	858 (53.5)	202 (23.7)	649 (76.3)	**0.001**
	Living without a partner	746 (46.5)	233 (31.5)	506 (68.5)	
Self-perceived health status	Bad or very bad	406 (25.0)	111 (27.5)	293 (72.5)	0.163
	Satisfactory	854 (52.6)	218 (25.8)	628 (74.2)	
	Good or very good	363 (22.4)	112 (31.1)	248 (68.9)	
Long-term illness	Yes	1245 (77.1)	321 (26.0)	914 (74.0)	**0.015**
	No	369 (22.9)	119 (32.4)	248 (67.6)	

Bold values indicate statistical significance (*p* < 0.05) based on chi-square test.

**Table 2:** Comparison of eating habits for older people with and without overnutrition.

Variable Name	Category	Body Weight		*p* Value
		BMI < 25	BMI ≥ 25	
Fruit consumption per week	Every day	309 (26.0)	880 (74.0)	**0.031**
	4–6 times a week	47 (27.8)	122 (72.2)	
	Less than 3 times a week	86 (34.1)	166 (65.9)	
Vegetable (except potato) consumption per week	Every day	217 (24.9)	655 (75.1)	**0.045**
	4–6 times a week	95 (31.5)	207 (68.5)	
	Less than 3 times a week	127 (29.5)	304 (70.5)	
Water consumption per day	At least 2 L	147 (21.6)	533 (78.4)	**<0.001**
	1–1.5 L	148 (28.5)	372 (71.5)	
	1 L or less	147 (35.7)	265 (64.3)	
Fruit juice consumption per week	Every day	43 (33.3)	86 (66.7)	**0.037**
	4–6 times a week	28 (37.3)	47 (62.7)	
	Less than 3 times a week	370 (26.5)	1028 (73.5)	
Sugary and soft drinks per week	Every day	33 (34.0)	64 (66.0)	0.306
	4–6 times a week	11 (25.6)	32 (74.4)	
	Less than 3 times a week	394 (26.9)	1069 (73.1)	
Sugar-free and diet drinks per week	More than once a week	42 (22.3)	146 (77.7)	0.091
	Less than once a week	399 (28.2)	1016 (71.8)	
Sweetener for hot drinks (coffee, tea)	Natural (sugar, honey)	253 (30.5)	577 (69.5)	**0.002**
	Artificial	83 (21.7)	299 (78.3)	
Sweets and desserts consumption	More than once a day	130 (28.9)	319 (71.1)	0.377
	Less than once a day	311 (26.8)	851 (73.2)	
Red meat consumption	More than 4 times a week	35 (24.6)	107 (75.4)	0.300
	1–3 times a week	152 (25.8)	437 (74.2)	
	Less than once a week	254 (29.0)	622 (71.0)	
White meat consumption	More than 4 times a week	79 (29.9)	185 (70.1)	0.081
	1–3 times a week	244 (25.4)	716 (74.6)	
	Less than once a week	119 (30.8)	267 (69.2)	
Processed meat consumption	More than 4 times a week	183 (27.3)	486 (72.7)	0.708
	1–3 times a week	138 (26.4)	385 (73.6)	
	Less than once a week	121 (28.8)	299 (71.2)	
Fish and seafood consumption	More than 4 times a week	9 (28.1)	23 (71.9)	0.422
	1–3 times a week	19 (21.4)	70 (78.6)	
	Less than once a week	412 (27.7)	1074 (72.3)	
Dairy product consumption	More than 4 times a week	285 (26.9)	774 (73.1)	0.195
	1–3 times a week	99 (26.3)	278 (73.7)	
	Less than once a week	58 (33.1)	117 (66.9)	
Salt consumption	Never or low salt use	331 (28.9)	816 (71.1)	**0.038**
	Moderate or high salt use	109 (23.7)	350 (76.3)	

Bold values indicate statistical significance (*p* < 0.05) based on chi-square test.

**Table 3:** Comparison of lifestyle factors for older people with and without overnutrition.

Variable Name	Category	Body Weight		*p* Value
		BMI < 25	BMI ≥ 25	
Alcohol consumption	Heavy	10 (14.5)	59 (85.5)	**0.044**
	Moderate	93 (26.8)	254 (73.2)	
	Rare or never	336 (28.2)	855 (71.8)	
Smoking status	Active	92 (44.2)	116 (55.8)	**<0.001**
	Quit	96 (21.9)	343 (78.1)	
	Never smoked	251 (26.2)	709 (73.8)	
General characteristics of daily physical activity	Mostly sitting or no movement	170 (27.5)	448 (72.5)	0.996
	Mostly standing	30 (28.3)	76 (71.7)	
	Mostly walking or moderate	229 (27.2)	613 (72.8)	
	Mostly heavy physical work	8 (27.6)	21 (72.4)	
Number of days walked for at least 10 min a week	Do not walk	88 (27.5)	232 (72.5)	0.495
	1–3 days	78 (24.9)	235 (75.1)	
	4–7 days	274 (28.4)	692 (71.6)	
Number of days cycled for at least 10 min a week	Do not cycle	322 (27.6)	885 (72.4)	0.930
	1–3 days	49 (26.6)	135 (73.4)	
	4–7 days	65 (26.6)	179 (73.4)	
Number of days performing sports for at least 10 min a week	Do not perform sports	310 (26.0)	883 (74.0)	**0.026**
	1–3 days	51 (26.8)	139 (73.2)	
	4–7 days	75 (34.9)	140 (65.1)	
Number of days performing muscle strengthening exercises for at least 10 min a week	No exercise	352 (26.8)	961 (73.2)	0.101
	1–3 days	33 (23.6)	107 (76.4)	
	4–7 days	48 (34.3)	92 (65.7)	
Sleep disturbances occurred in last 2 weeks	Never	204 (26.8)	558 (73.2)	0.775
	In a few days	149 (27.2)	399 (72.8)	
	More than a week	40 (31.2)	88 (68.8)	
	Almost every day	44 (27.2)	123 (73.6)	

Bold values indicate statistical significance (*p* < 0.05) based on chi-square test.

**Table 4:** Logistic regression results for overnutrition in the elderly population in Hungary.

Variable	Category/Level	OR	95% CI	*p*-Value
Gender	Male (Reference)			
	Female	0.81	0.57–1.14	0.236
Income levels	Lower than average (Reference)			
	Average	0.90	0.63–1.27	0.555
	Higher than average	0.76	0.53–1.07	0.124
Degree of urbanization	Urban (Reference)			
	Suburban	1.24	0.78–1.95	0.350
	Rural	0.85	0.57–1.28	0.459
	Remote	1.16	0.75–1.80	0.494
Partner status	Living with a partner (Reference)			
	Living without a partner	0.78	0.57–1.06	0.118
Long-term illness	Yes (Reference)			
	None	0.80	0.58–1.12	0.204
Fruit consumption	Every day (Reference)			
	4–6 times a week	0.98	0.62–1.55	0.791
	Less than 3 times a week	0.68	0.46–1.01	0.062
Vegetable consumption	Every day (Reference)			
	4–6 times/week	0.83	0.57–1.22	0.360
	1–3 times/week	0.92	0.65–1.30	0.651
Water intake	More than 2 L (Reference)			
	1.5–2 L	0.68	0.48–0.95	**0.025**
	1–1.5 L	0.47	0.33–0.65	**<0.001**
Fruit juice intake	Every day (Reference)			
	4–6 times/week	0.95	0.43–1.98	0.860
	1–3 times/week	1.43	0.86–2.38	0.160
Sweetener use for tea or coffee	Natural (Reference)			
	Artificial	1.54	1.13–2.11	**0.006**
Salt use	Never or low salt use			
	Moderate or high salt use	1.45	1.06–1.99	**0.020**
Smoking status	Active (Reference)			
	Quit	2.32	1.49–3.61	**<0.001**
	Never smoked	2.56	1.73–3.77	**<0.001**
Alcohol use	Heavy drinker (Reference)			
	Moderate drinker	0.26	0.09–0.80	**<0.019**
	Rare drinker	0.25	0.08–0.75	**<0.018**
Days of ten-minute sport per week	Do not perform sports (Reference)			
	1–3 days	0.92	0.58–1.43	0.714
	4–7 days	0.74	0.48–1.13	0.171

Bold values indicate statistical significance (*p* < 0.05). Odds ratios are adjusted for variables in the model. AUC = 0.6708 (95% CI 0.637–0.705).

## Data Availability

The data analyzed in this study are subject to the following licenses/restrictions: The data presented in this study are available upon request from Hungarian Central Statistical Office who performed and supervised the data collection. Requests to access these datasets should be directed to Hungarian Central Statistical Office, www.ksh.hu/?lang=en (accessed on 1 April 2025).

## References

[1] Whaley-Connell A., Pulakat L., DeMarco V.G., Hayden M.R., Habibi J., Henriksen E.J., Sowers J.R. (2011). Overnutrition and the Cardiorenal Syndrome: Use of a Rodent Model to Examine Mechanisms. Cardiorenal Med..

[2] Obesity and Overweight. https://www.who.int/news-room/fact-sheets/detail/obesity-and-overweight.

[3] OECD, European Union (2022). Health at a Glance: Europe 2022: State of Health in the EU Cycle. Health at a Glance: Europe.

[4] Dee A., Kearns K., O’neill C., Sharp L., Staines A., O’dwyer V., Fitzgerald S., Perry I.J. (2014). The direct and indirect costs of both overweight and obesity: A systematic review. BMC Res. Notes.

[5] Ulambayar B., Ghanem A.S., Tóth Á., Nagy A.C. (2025). Impact of Physical Activity and Dietary Habits on Mental Well-Being in Patients with Diabetes Mellitus. Nutrients.

[6] Jaggi P. (2020). Ageing Population of Urban India & Psychological Well-being Issues. Int. J. Soc. Sci..

[7] Ageing and Health. https://www.who.int/news-room/fact-sheets/detail/ageing-and-health.

[8] Moscatelli F., De Maria A., Marinaccio L.A., Monda V., Messina A., Monacis D., Toto G., Limone P., Monda M., Messina G. (2023). Assessment of Lifestyle, Eating Habits and the Effect of Nutritional Education among Undergraduate Students in Southern Italy. Nutrients.

[9] Roberts S.B., Rosenberg I. (2006). Nutrition and Aging: Changes in the Regulation of Energy Metabolism with Aging. Physiol. Rev..

[10] Boulos C., Salameh P., Barberger-Gateau P. (2017). Social isolation and risk for malnutrition among older people. Geriatr. Gerontol. Int..

[11] Story M., Kaphingst K.M., Robinson-O’Brien R., Glanz K. (2008). Creating Healthy Food and Eating Environments: Policy and Environmental Approaches. Annu. Rev. Public Health.

[12] Tripathi S., Banerjee D., Murry B. (2023). Estimation of over nutrition among the elderly population of India: A systematic review. Int. J. Res. Med. Sci..

[13] Leung D.Y.P., Cheng H.-L., Tyrovolas S., Tang A.S.K., Liu J.Y.W., Tse M.M.Y., Lai C.K.Y., Molassiotis A. (2021). Magnitude, Temporal Trends, and Inequalities in the DALYs and YLDs of Nutritional Deficiency among Older Adults in the Western Pacific Region: Findings from the Global Burden of Disease Study 1990–2019. Nutrients.

[14] Mathur P., Pillai R. (2019). Overnutrition: Current scenario & combat strategies. Indian J. Med. Res..

[15] Xin Y., Ren X. (2021). The Impact of Family Income on Body Mass Index and Self-Rated Health of Illiterate and Non-illiterate Rural Elderly in China: Evidence from a Fixed Effect Approach. Front. Public Health.

[16] Wang G., Hao Y., Ma J. (2024). Family Income Level, Income Structure, and Dietary Imbalance of Elderly Households in Rural China. Foods.

[17] Okumiya K., Ishine M., Kasahara Y., Wada T., Sakamoto R., Kosaka Y., Ishimoto Y., Hirosaki M., Kimura Y., Fujisawa M. (2011). The effects of socioeconomic globalization on health and aging in highlanders compared to lowlanders in Yunnan, China, and Kochi, Japan. Ecol. Res..

[18] Swinburn B.A., Sacks G., Hall K.D., McPherson K., Finegood D.T., Moodie M.L., Gortmaker S.L. (2011). The global obesity pandemic: Shaped by global drivers and local environments. Lancet.

[19] Kanter R., Caballero B. (2012). Global Gender Disparities in Obesity: A Review. Adv. Nutr. Int. Rev. J..

[20] Wardle J., Parmenter K., Waller J. (2000). Nutrition knowledge and food intake. Appetite.

[21] Drewnowski A., Specter S. (2004). Poverty and obesity: The role of energy density and energy costs. Am. J. Clin. Nutr..

[22] Rurik I., Sandholzer H. (2009). Obesity among Hungarian elderly. Acta Aliment..

[23] Soós R., Bakó C., Gyebrovszki Á., Gordos M., Csala D., Ádám Z., Wilhelm M. (2024). Nutritional Habits of Hungarian Older Adults. Nutrients.

[24] OECD, European Observatory on Health Systems and Policies (2023). Hungary: Country Health Profile 2023. State of Health in the EU.

[25] Popkin B.M., Adair L.S., Ng S.W. (2012). Global nutrition transition and the pandemic of obesity in developing countries. Nutr. Rev..

[26] Brown T., Moore T.H., Hooper L., Gao Y., Zayegh A., Ijaz S., Elwenspoek M., Foxen S.C., Magee L., O’Malley C. (2019). Interventions for preventing obesity in children. Cochrane Database Syst. Rev..

[27] Perez-Martinez P., Phillips C., Delgado-Lista J., Garcia-Rios A., Lopez-Miranda J., Perez-Jimenez F. (2014). Nutrigenetics, Metabolic Syndrome Risk and Personalized Nutrition. Curr. Vasc. Pharmacol..

[28] European Health Interview Survey―Methodology. https://ec.europa.eu/eurostat/statistics-explained/index.php?title=European_health_interview_survey_-_methodology.

[29] Obesity. https://www.who.int/health-topics/obesity.

[30] Healthy Diet. https://www.who.int/news-room/fact-sheets/detail/healthy-diet.

[31] (2023). Stata Statistical Software.

[32] Leitão C., Mignano A., Estrela M., Fardilha M., Figueiras A., Roque F., Herdeiro M.T. (2022). The Effect of Nutrition on Aging—A Systematic Review Focusing on Aging-Related Biomarkers. Nutrients.

[33] Hasan M., Sutradhar I., Shahabuddin A., Sarker M. (2017). Double Burden of Malnutrition among Bangladeshi Women: A Literature Review. Cureus.

[34] Kim I.-H., Chun H., Kwon J.-W. (2011). Gender Differences in the Effect of Obesity on Chronic Diseases among the Elderly Koreans. J. Korean Med. Sci..

[35] Wells J.C., Sawaya A.L., Wibaek R., Mwangome M., Poullas M.S., Yajnik C.S., Demaio A. (2020). The double burden of malnutrition: Aetiological pathways and consequences for health. Lancet.

[36] Ferdous T., Kabir Z.N., Wahlin Å., Streatfield K., Cederholm T. (2009). The multidimensional background of malnutrition among rural older individuals in Bangladesh―A challenge for the Millennium Development Goal. Public Health Nutr..

[37] Selvamani Y., Singh P. (2018). Socioeconomic patterns of underweight and its association with self-rated health, cognition and quality of life among older adults in India. PLoS ONE.

[38] Encalada-Torres J., Abril-Ulloa V., Wong S., Alvarado-Romero S., Bedoya-Ortega M., Encalada-Torres L. (2022). Socioeconomic Status and Nutritional Status as Predictors of Food Insecurity in Older Adults: A Case Study from Southern Ecuador. Int. J. Environ. Res. Public Health.

[39] Nazri N.S., Vanoh D., Leng S.K. (2020). Malnutrition, low diet quality and its risk factors among older adults with low socio-economic status: A scoping review. Nutr. Res. Rev..

[40] Vagnetti R., Camp N., Story M., Ait-Belaid K., Mitra S., Davis S.F., Meese H., Zecca M., Di Nuovo A., Magistro D. (2024). Social Robots and Sensors for Enhanced Aging at Home: Mixed Methods Study with a Focus on Mobility and Socioeconomic Factors. JMIR Aging.

[41] Ishida Y., Yoshida D., Honda T., Hirakawa Y., Shibata M., Sakata S., Furuta Y., Oishi E., Hata J., Kitazono T. (2020). Influence of the Accumulation of Unhealthy Eating Habits on Obesity in a General Japanese Population: The Hisayama Study. Nutrients.

[42] Rousset S., Mirand P.P., Brandolini M., Martin J.-F., Boirie Y. (2003). Daily protein intakes and eating patterns in young and elderly French. Br. J. Nutr..

[43] Lasheras C., González C., Patterson A.M., Fernández S. (1998). Food Habits and Anthropometric Measurements in a Group of Independent and Institutionalized Elderly People in Spain. J. Nutr. Sci. Vitaminol..

[44] Laszlo N., Aboul-Enein B.H., Bernstein J., Kruk J. (2017). Addressing Cardiovascular Disease Risk in HungarianAmerican Populations: A Cultural Exploration of Transdisciplinary Health Promotion. Cent. Eur. J. Sport Sci. Med..

[45] Donini L.M., Poggiogalle E., Piredda M., Pinto A., Barbagallo M., Cucinotta D., Sergi G. (2013). Anorexia and Eating Patterns in the Elderly. PLoS ONE.

[46] Rautiainen S., Wang L., Lee I.-M., Manson J.E., Buring J.E., Sesso H.D. (2015). Higher Intake of Fruit, but Not Vegetables or Fiber, at Baseline Is Associated with Lower Risk of Becoming Overweight or Obese in Middle-Aged and Older Women of Normal BMI at Baseline. J. Nutr..

[47] Guo Q., Fang H., Zhao L., Ju L., Xu X., Yu D. (2023). Level of Fruit and Vegetable Intake and Its Relationship with Risk for Malnutrition in China’s Adult Labor Force: China Nutrition and Health Surveillance, 2015–2017. Nutrients.

[48] Drouin-Chartier J.-P., Zheng Y., Li Y., Malik V., Pan A., Bhupathiraju S.N., Tobias D.K., Manson J.E., Willett W.C., Hu F.B. (2019). Changes in Consumption of Sugary Beverages and Artificially Sweetened Beverages and Subsequent Risk of Type 2 Diabetes: Results from Three Large Prospective U.S. Cohorts of Women and Men. Diabetes Care.

[49] Heo G.Y., Koh H.B., Park J.T., Han S.H., Yoo T.-H., Kang S.-W., Kim H.W. (2024). Sweetened Beverage Intake and Incident Chronic Kidney Disease in the UK Biobank Study. JAMA Netw. Open.

[50] Kan J., Wang D.-C., Chang Y., Jiang Z.-H., Jiang X.-M., Xie H., Jia X.-X., Chen M.-X., Gu Y. (2024). Associations of artificial sweetener intake with cardiometabolic disorders and mortality: A population-based study. Br. J. Nutr..

[51] Debras C., Deschasaux-Tanguy M., Chazelas E., Sellem L., Druesne-Pecollo N., Esseddik Y., de Edelenyi F.S., Agaësse C., De Sa A., Lutchia R. (2023). Artificial Sweeteners and Risk of Type 2 Diabetes in the Prospective NutriNet-Santé Cohort. Diabetes Care.

[52] Sun T., Yang J., Lei F., Huang X., Liu W., Zhang X., Lin L., Sun L., Xie X., Zhang X.-J. (2024). Artificial sweeteners and risk of incident cardiovascular disease and mortality: Evidence from UK Biobank. Cardiovasc. Diabetol..

[53] Shil A., Zhang J., Chichger H. (2023). Investigating the use and awareness of artificial sweeteners among diabetic patients in Bangladesh. PLoS ONE.

[54] He F.J., Li J., MacGregor G.A. (2013). Effect of longer term modest salt reduction on blood pressure: Cochrane systematic review and meta-analysis of randomised trials. BMJ.

[55] Yoshida Y., Kosaki K., Sugasawa T., Matsui M., Yoshioka M., Aoki K., Kuji T., Mizuno R., Kuro-O M., Yamagata K. (2020). High Salt Diet Impacts the Risk of Sarcopenia Associated with Reduction of Skeletal Muscle Performance in the Japanese Population. Nutrients.

[56] Zhou L., Stamler J., Chan Q., Van Horn L., Daviglus M.L., Dyer A.R., Miura K., Okuda N., Wu Y., Ueshima H. (2019). Salt intake and prevalence of overweight/obesity in Japan, China, the United Kingdom, and the United States: The INTERMAP Study. Am. J. Clin. Nutr..

[57] Takase H., Takeuchi Y., Fujita T., Ohishi T. (2023). Excessive salt intake reduces bone density in the general female population. Eur. J. Clin. Investig..

[58] Kojima G., Iliffe S., Walters K. (2015). Smoking as a predictor of frailty: A systematic review. BMC Geriatr..

[59] Xu J., Tian G., Zhang T., Zhang H., Liu J., Shi Q., Sun J., Wang H., Zhang B., Wu Q. (2022). Assessing the income-related inequality in obesity among the elderly in China: A decomposition analysis. Front. Public Health.

[60] Hsieh T.-H., Lee J.J., Yu E.W.-R., Hu H.-Y., Lin S.-Y., Ho C.-Y. (2020). Association between obesity and education level among the elderly in Taipei, Taiwan between 2013 and 2015: A cross-sectional study. Sci. Rep..

[61] Lee G., Park J., Oh S.-W., Joh H.-K., Hwang S.-S., Kim J., Park D. (2017). Association between Body Mass Index and Quality of Life in Elderly People over 60 Years of Age. Korean J. Fam. Med..

[62] Shin M.-H., Kweon S.-S., Choi J.-S., Rhee J.-A., Nam H.-S., Jeong S.-K., Park K.-S., Ryu S.-Y., Choi S.-W., Kim B.-H. (2013). Average Volume of Alcohol Consumed, Drinking Patterns, and Metabolic Syndrome in Older Korean Adults. J. Epidemiol..

[63] Destro J.S.F., Marin M.J.S., Otani M.A.P., Selleti J.D.D.N., Higa E.D.F.R. (2022). Experiences of alcohol-dependent elderly: Grounded theory. Rev. Esc. Enferm. USP.

[64] Amadea V.C., Dinata M., Sarvasti D. (2022). Correlation of Physical Activity with Total Cholesterol Ratio to High-Density Lipoprotein in Hypertension Elderly at Puskesmas Gedangan Sidoarjo. J. Widya Med. Jr..

[65] Sbardelotto M.L., Costa R.R., Malysz K.A., Pedroso G.S., Pereira B.C., Sorato H.R., Silveira P.C.L., Nesi R.T., Grande A.J., A Pinho R. (2019). Improvement in muscular strength and aerobic capacities in elderly people occurs independently of physical training type or exercise model. Clinics.

[66] Wang S., Ma W., Wang S.-M., Yi X. (2018). A Cross Sectional Examination of the Relation Between Depression and Frequency of Leisure Time Physical Exercise among the Elderly in Jinan, China. Int. J. Environ. Res. Public Health.

[67] Justine M., Azizan A., Hassan V., Salleh Z., Manaf H. (2013). Barriers to participation in physical activity and exercise among middle-aged and elderly individuals. Singap. Med. J..

[68] Rosinger A.Y. (2020). Biobehavioral variation in human water needs: How adaptations, early life environments, and the life course affect body water homeostasis. Am. J. Hum. Biol..

[69] Zhang J., Ren Z., Zhang Q., Zhang R., Zhang C., Liu J. (2022). Lower hydration status increased diabetic retinopathy among middle-aged adults and older adults: Results from NHANES 2005–2008. Front. Public Health.

[70] Padrão P., Sousa A.S., Guerra R.S., Álvares L., Santos A., Borges N., Afonso C., Amaral T.F., Moreira P. (2017). A Cross-Sectional Study on the Association between 24-h Urine Osmolality and Weight Status in Older Adults. Nutrients.

[71] Queirós C., Machado F.B., Barros D., Sampaio J., Sampaio A., Barros R., Moreira P., Ribeiro Ó., Carvalho J., Padrão P. (2023). Urinary Hydration Biomarkers and Water Sources in Older Adults with Neurocognitive Disorder. Nutrients.

